# Resilience and traumatic stress among Latinx english language learners: a cross-sectional study of students from an urban school district

**DOI:** 10.1186/s12889-025-23105-4

**Published:** 2025-07-15

**Authors:** Roya Ijadi-Maghsoodi, Sara Rahmanian Koushkaki, Alexandra Klomhaus, Hilary Aralis, Angela Venegas-Murillo, Lauren Marlotte, Sameera Siddiqi, Kungeun Lee, Shirley A. De La Cruz, Sheryl Kataoka

**Affiliations:** 1 Department of Psychiatry and Biobehavioral Sciences, David Geffen School of Medicine at UCLA, Los Angeles, CA USA; 2 UCLA Division of Population Behavioral Health, Jane and Terry Semel Institute for Neuroscience, Human Behavior at UCLA, Los Angeles, CA USA; 3 Center for the Study of Healthcare Innovation, Implementation & Policy (CSHIIP), VA Greater Los Angeles Healthcare System, Los Angeles, CA USA; 4 Division of Biostatistics, Department of Public Health Sciences at UC Davis, Davis, CA USA; 5 Department of Pediatrics, Charles R. Drew University of Medicine and Science, Los Angeles, CA USA

**Keywords:** Psychological resilience, Hispanic or Latino, English learners, Traumatic stress

## Abstract

**Background:**

Latinx students in the United States can face stressors and structural inequities that can lead to poor academic and mental health outcomes. They comprise 76% of the English Language Learner (ELL) population, yet little is known about the relationship between ELL status and traumatic stress and resilience outcomes among these Latinx students. We sought to see if resilience differs between ELL vs. non-ELL Latinx students, and if traumatic stress risk modifies the association between ELL designation and resilience among Latinx students to inform culturally relevant school resilience interventions and school-wide approaches for this population.

**Methods:**

We analyzed deidentified school district administrative and survey data from a convenience sample of mostly Latinx 6-12th graders from one large, urban U.S. school district. We restricted our sample to Latinx students, resulting in a sample of 4,950 students attending 91 middle and high schools. We constructed linear regression models to understand differences in internal and external resilience based on ELL status, traumatic stress risk, and their interaction.

**Results:**

Among students with low traumatic stress risk, ELL students had worse self-efficacy but better problem solving than their non-ELL peers. When considering students with high traumatic stress risk, ELL students had better problem solving, self-awareness, perceived school support, and total internal assets, relative to non-ELL students.

**Conclusions:**

Latinx students designated as ELL may demonstrate resilience despite adversity; these resilience assets may be further amplified among the subset of students at high risk for traumatic stress. Our findings may inform school resilience interventions and school supports for ELL Latinx students.

**Supplementary Information:**

The online version contains supplementary material available at 10.1186/s12889-025-23105-4.

## Introduction

As international migration to the U.S. continues to increase for reasons including seeking better economic, educational, and living opportunities, and fleeing conflict, trauma, and persecution, schools remain essential systems to welcome and meet the needs of students who are immigrants or children of immigrants [1, 2]. In the United States (U.S.), the English Language Learner (ELL) population—consisting of students born outside of and within the U.S—reached 5 million students in 2020 [3]. Students are designated as English Language Learners when their school identifies them with challenges in speaking, reading, writing, or understanding the English language which may prevent them from meeting state academic and achievement standards. Once designated as English Language Learners, students can qualify to receive language instruction as part of their educational program as determined by the State Educational Agency [4]. Although students designated as English Language Learners (hereafter referred to as ELL students) have many strengths, ELL students are at risk for greater educational and social-emotional challenges compared to non-ELL students [5]. Nationally, ELL students perform lower on reading achievement tests compared to non-ELL students. While the achievement gap for ELL students in the U.S. decreased from 1998 to 2022 for both fourth and eighth graders, it worsened for twelfth grade students [6]. Academic achievement is a critical student outcome for ELL students; low academic achievement is tied to later health, mental health, and economic disparities [7]. In the U.S, more than one-quarter of all public PK-12 students are Latinx, and Latinx students account for 76% of ELL students [3, 8]. Although the needs of all ELL students are important, in this manuscript we focus specifically on Latinx ELL students in the U.S.

Latinx students—regardless of ELL status—can experience heightened mental health and academic challenges related to stressors of trauma, racism, police violence, housing insecurity, poverty, school biases, lack of culturally tailored interventions, and other systemic inequities that can affect mental health and academic outcomes [9–13]. ELL Latinx students may experience additional stressors particularly related to their ELL status, including navigating language barriers for themselves and their caregivers [14]. Stressors for Latinx ELL students who are immigrants themselves or children of immigrants include immigration trauma (e.g. potential family separation and traumatic migration experiences), trauma or persecution within their country of origin, fears over immigration status, barriers accessing adequate health and mental health care, discrimination, and intergenerational conflict [15–18]. Prior studies have demonstrated that Latinx ELL students report increased levels of acculturative stress and discrimination compared to non-ELL Latinx youth [5, 19]. Spanish speaking ELL students have reported higher social-emotional challenges compared to their English monolingual and Asian language ELL peers, which is thought to partially explain their later lower academic achievement [20].

Although there is a lack of mental health prevalence studies among Latinx ELL students in the U.S., Latinx ELL students are thought to be at greater risk for mental health challenges with less identification of need. For example, a meta-analysis found that ELL students were significantly under-represented in the emotional behavioral disturbance category of special education [21]. Research demonstrates a greater lifetime prevalence of mood and anxiety disorders among Latinx youth compared to white youth in the U.S, along with lower likelihood of receiving mental health services among first generation Latinx youth [22]. Studies with immigrant youth and smaller samples of Latinx ELL students also indicate risk of mental health challenges [23, 24]. Research with recent immigrant children found that 32% of the sample reported clinical PTSD symptoms, and 16% reported clinical depressive symptoms [23]. A small study of mostly Latinx ELL students found that average baseline depression and anxiety scores were above the clinical cut-off level [24]. One study examining post-traumatic stress disorder (PTSD) symptoms among Latinx immigrant youth found that female youth reported higher level of PTSD symptoms than males and that perceived discrimination was associated with higher PTSD symptoms among females in the sample [25].

### Literature Review-Resilience

Despite exposure to stressors and trauma, youth can exhibit considerable resilience—the capacity for successful adaptation to adversity [26]. Luthar and Cicchetti describe resilience as “positive adaptation,” successfully meeting appropriate developmental tasks, occurring in the midst of “adversity” or negative life experiences known to be associated with risks to positive youth development. For older youth, this includes forming appropriate peer relationships, and school functioning [27]. For operationalizing and measuring resilience among youth, researchers categorize resilience as comprised of *internal assets* or personal strengths such as problem-solving and self-efficacy, and *external resources* such as family, school, and community supports [28, 29]. Greater levels of resilience are associated with less adverse outcomes and trauma-related symptoms [30, 31]. Family and peer supports, participation in school activities, cultural factors, and community supports are all factors contributing to resilience among Latinx youth that are linked to positive academic and mental health outcomes [32–34]. Research examining resilient versus non-resilient Latinx ELL students found that higher self-efficacy and a supportive home environment contributed to increased academic resilience in Latinx ELL students [35]. Research has also demonstrated social emotional outcomes strengths among bilingual learners. One study of socio-emotional trajectories among elementary age Latinx children found that children characterized as Fluent Bilingual and Non-English Dominant Bilingual had the strongest trajectories in regard to measures of approach to learning, self-control, and interpersonal skills, with lower externalizing and internalizing problems by fifth grade, compared to monolingual and English-Dominant Bilingual peers [36]. However, Castro-Olivo and colleagues found that among secondary school Latinx ELL students, the longer students participated in English Language Development programs, the lower they scored on measures of social-emotional resiliency, leading authors to call for social-emotional resilience-building programs to be integrated into the curriculum for ELL students [37]. Although research is growing, there is still a gap in understanding the relationship between traumatic stress and resilience characteristics specifically among Latinx ELL students and how to bolster resilience in this population.

Researchers have built upon the foundational frameworks for risk and resilience to develop specific conceptual models for understanding risk and resilience among immigrant youth [27, 38]. Motti-Stefanidi and colleagues’ developed an integrative multi-level framework to understand immigrant youth adaptation, consisting of an individual level, level of interaction including schools, ethnic group, and parents and families, and societal level [39]. Utilizing the same multi-level approach, Suárez-Orozco and colleagues’ integrative risk and resilience model for immigrant origin children and youth (children and youth with at least one foreign-born parent) delineate four levels of contexts affecting immigrant-origin children and youth framed by the authors as potential risks or resources for adaptation, including contexts related to undocumented and refugee youth. These levels include (1) global forces (e.g. wars and violence), (2) Political and Social Contexts of Reception (e.g. national and state immigration policies), (3) Microsystems (e.g. neighborhoods, schools), and (4) Individual Level (e.g. race-ethnicity, gender, exposure to traumatic events). Suárez-Orozco and colleagues describe positive adaptation of immigrant-origin children and youth across the areas of (1) developmental tasks (e.g. having close friends, being successful in school), (2) psychological adjustment (e.g. having self-esteem, lack of PTSD symptoms), and (3) acculturative tasks (e.g. acquiring cultural skills and knowledge of the receiving community while learning and practicing values of the family’s culture of origin, and preserving ethnic identities) [18]. Regarding the role of schools in the model, the authors describe how schools can support immigrant-origin children and youth, including schools that are well-integrated, that support identity development and acculturative tasks, and promote supportive relationships with school staff and peers [18]. Although prior conceptual frameworks, including Motti-Stefanidi and colleagues’ multi-level framework, are informative, Suárez-Orozco’s integrative model is most applicable to the immigrant and ELL student population in the U.S., which includes undocumented and refugee students [18, 39].

As highlighted by Suárez -Orozco’s integrative risk and resilience model, and the WHO and UN agencies (see World Health Organization’s Health-Promoting Schools Framework), schools are increasingly recognized world-wide as critical settings to improve social-emotional wellbeing and mental health promotion for students, particularly for students exposed to trauma and social inequities [18, 40, 41]. Schools are adopting whole-school and classroom level approaches to promote resilience that can mitigate against the effects of trauma and social inequities, to improve academic and mental health outcomes. There is increasing evidence that social-emotional learning (SEL), and resilience interventions should be culturally tailored for student populations, including Latinx ELL students [14, 42, 43]. However, despite calls from experts to tailor school interventions to the unique stressors and needs of Latinx ELL students, there is a paucity of interventions culturally adapted to promote resilience and support positive coping with stressful experiences among Latinx ELL students [14]. Given potentially differing life experiences and needs among Latinx ELL students than general student populations, there is a need to further understand stressors and resilience factors among this population to inform how to best support all students in a school setting.

Due to the growing number of Latinx ELL students in the U.S., and the need to understand the relationship between trauma and resilience among student populations to best inform school practices and interventions, our study focused on two main questions: (1) Does resilience differ between ELL vs. non-ELL Latinx students? and (2) Does traumatic stress risk modify the association between ELL designation and resilience among Latinx students? The first question builds upon research examining resilience among ELL vs. non-ELL Latinx students by particularly examining external resilience in the school environment, and the second question adds to the literature by examining the role of traumatic stress risk within the Latinx ELL population [35]. Findings from these questions can inform educational and school mental health services and policies for Latinx ELL students to improve their academic and social-emotional outcomes. This could involve tailoring services through school mental health programs, classroom-level interventions to strengthen resilience factors, and whole-school supports, including culturally adapted efforts to foster internal resilience asserts and strengthen external supports across the school setting.

To investigate these questions, we used administrative data from a large urban school district in the western region of the U.S., that serves mostly under-resourced and minoritized students, including a high proportion of Latinx youth. Our study was informed by underlying resilience frameworks, including Luthar and Cichetti’s construct of resilience, and the conceptualization of internal and external resilience assets contributing to student positive development described by Benard and Slade, while focusing on the specific microsystem of schools as described by Suárez-Orozco [18, 27, 29]. This district prioritizes bolstering resilience and wellbeing among students and parents through efforts including measuring student resilience and risk of traumatic stress and providing multi-level prevention and supports to address student trauma and resilience. While we note several limitations to our design, including utilizing a convenience sample of students and a cross-sectional design (discussed further in the limitation section), given the limited body of research in this area for Latinx ELL students, our findings can help address this gap and inform potential school interventions.

## Methods

### Design

We employed a cross-sectional, observational study design utilizing deidentified administrative survey data collected by the District that included traumatic stress risk and resilience measures among a convenience sample of 6th -12th grade (middle and high school) Latinx students. Despite drawbacks to using a convenience sample (e.g. generalizability), one benefit that we considered is the reduced burden on the district in using data that they were already collecting as part of the district’s prevention program. The ethical consideration of this design included avoiding the conduct of research (i.e. collecting extra survey data) that may not directly benefit participants. In addition to the survey data, the administrative data contained demographic characteristics and academic outcomes for the sample of students who completed surveys. Thus, this research was conducted entirely through secondary data analysis of district administrative data; the research team was not involved in student recruitment or data collection. The district collected all surveys themselves as part of their usual care support and followed district protocols. This study was approved by the District’s research review committee and met criteria for an exemption from IRB review by the University Internal Review Board.

During the 2017–2018 academic school year, as part of their efforts to focus on prevention and wellbeing, the District administered a risk and resilience survey to students in middle and high school, toward the beginning of the Fall and Spring semesters. The survey was adapted by the school mental health team from a population-level assessment tool. This adapted survey was used to support the work of the school mental health team and was often administered in a classroom setting prior to delivery of a classroom-based prevention program. District psychiatric social workers (PSWs) administered the survey to students in English and Spanish. Students filled out the survey on an online platform which was typically completed within a single one-hour class session. The District and school leadership selected schools and classrooms in which to administer the survey based on factors such as administrator support for these efforts, and feasibility of conducting the surveys and support programs, including staff availability. This study relied on the deidentified administrative data collected by the district, with schools and classrooms selected by school staff.

### Participants

Students included in this study attended a large, urban school district in the western U.S. 84% of students were living in poverty. Approximately three-quarters of the District’s enrolled students identified as Latinx (mainly of Mexican descent), and about one-quarter of students were designated as ELL, with the primary language as Spanish. When examining the District’s sociodemographic characteristics, 55% of ELL students are male, compared to 50.4% of non-ELL students, and 22% of ELL students are receiving special education services compared to 9.6% of non-ELL students. These district characteristics are fairly comparable to our sample (See Table 1).

**Table 1:** Characteristics describing the sample of ELL and Non-ELL Latinx students

Student characteristics (*N* = 4950)	ELL (*N* = 836)	Non-ELL (*N* = 4114)	Total
	*N* (%)		
**Gender** ^*^			
Female	370 (44.26)	2005 (48.74)	2375 (47.98)
Male	466 (55.74)	2109 (51.26)	2575 (52.02)
**Grade** ^***^			
Middle School (6–8)	405 (48.44)	1313 (31.92)	1718 (34.71)
High School (9–12)	431 (51.56)	2801 (68.08)	3232 (65.29)
**Special Education** ^***^			
Not Eligible	593 (70.93)	3799 (92.34)	4392 (88.73)
Eligible	243 (29.07)	315 (7.66)	558 (11.27)
**Traumatic Stress Risk**			
High Risk	217 (25.96)	1069 (25.98)	1286 (25.98)
Low Risk	585 (69.98)	2981 (72.46)	3566 (72.04)
Missing	34 (4.07)	64 (1.56)	98 (1.98)
**School Attendance** ^*^			
Basic or Below	277 (33.13)	1233 (29.97)	1510 (30.51)
Proficient/Advanced	537 (64.23)	2849 (69.25)	3386 (68.40)
Missing	22 (2.63)	32 (0.78)	54 (1.09)
**Grade point average (GPA)** ^***^			
Not Passing	232 (27.75)	984 (23.92)	1216 (24.57)
Passing	293 (35.05)	2247 (54.62)	2540 (51.31)
Missing	311 (37.20)	883 (21.46)	1194 (24.12)
**Birth Country** ^1***^			
United States	595 (71.17)	3864 (93.92)	4459 (90.08)
Mexico	54 (6.46)	162 (3.94)	216 (4.36)
El Salvador	118 (14.11)	47 (1.14)	165 (3.33)
Guatemala	43 (5.14)	24 (0.58)	67 (1.35)
Honduras	16 (1.91)	9 (0.22)	25 (0.51)
Other^2^	10 (1.20)	8 (0.19)	18 (0.36)
**Internal Resilience** ^3^			
Self-Efficacy, Mean (SD)^***^	2.93 (0.61)	3.01 (0.56)	2.99 (0.57)
Empathy, Mean (SD)^***^	3.02 (0.73)	3.12 (0.72)	3.10 (0.72)
Problem Solving, Mean (SD)^***^	2.66 (0.87)	2.50 (0.89)	2.53 (0.89)
Self-Awareness, Mean (SD)^*^	3.10 (0.73)	3.17 (0.73)	3.16 (0.73)
Total Internal Assets, Mean (SD)	2.95 (0.56)	2.99 (0.52)	2.98 (0.53)
**External Resilience** ^4^			
Perceived School Support, Mean (SD)^*^	3.12 (0.70)	3.05 (0.72)	3.06 (0.72)

^*^p < 0.05, ^**^p < 0.01, ^***^p < 0.01 for Pearson chi-square test of independence for categorical variables, and for a two-sample t-test for continuous variables

^1^Chi-square test comparing United States vs. all other birth countries combined

^2^Other includes: Brazil, Colombia, Costa Rica, Cuba, Nicaragua, Peru, Philippines, Puerto Rico, Venezuela

^3^*N* = 4858 for Self-Efficacy, Problem-Solving; *N* = 4857 for Empathy; *N* = 4859 for Self-Awareness; *N* = 4856 for Total Internal Assets

^4^*N* = 4860 for Perceived School Support

Of the 6,750 6-12th grade students who completed the survey, the District also had information on ELL status for 5,777 (85.6%) of those students from their 2016–2017 academic record data. Among the 5,777 sample, we further restricted our sample to include only “Hispanic” students resulting in an analytical sample of 4,950 students (85.7%). These students attended 91 middle and high schools.

### Measures

#### Student characteristics

We obtained student demographic and academic data including gender, grade, race/ethnicity, birth country, special education eligibility, and ELL status (Table 1). The District identifies a student as an ELL student, based on an administered assessment, if the student does not demonstrate proficiencies in listening, speaking, reading, and writing in English sufficient to take part in the standard school program. The District has a history of serving ELL students and supporting newcomer students for over three decades. Prior-year academic indicators available included attendance rate and grade point average (GPA). Attendance rate reflected the percentage of days attended across the entire year. In accordance with the District’s attendance goal, we categorized student attendance as *Proficient/Advanced* if the attendance rate was ≥ 96% (0–7 absences) and as *Basic or Below* if the attendance rate was < 96% (> 7 absences). We categorized GPA as *Passing* if the GPA ≥ 2.00 and *Not Passing* if < 2.00. GPA was based on grades earned from both academic semesters in the prior year on a scale from 0.00 to 4.00. A non-negligible proportion of our sample had missing information because some students presumably attended a school for which GPA was not calculated in the prior year, for instance, elementary school.

#### Risk for traumatic stress

Risk of traumatic stress—reactions that may develop after a traumatic event that can affect daily functioning [44]—was measured using the Primary Care PTSD survey for DSM-IV (PC-PTSD) [45]. The PC-PTSD begins with a prompt referencing a traumatic event (“In your life, have you ever had any experience that was so frightening, horrible, or upsetting that, in the past month, you…”), followed by four yes/no” questions inquiring about PTSD symptoms. We characterized a response of “yes” to three or more items (total score of ≥ 3) as indicating *High Risk for Traumatic Stress*, and a total score of < 3 as indicating *Low Risk for Traumatic Stress*, consistent with prior studies among primary care and Latinx participants [45, 46]. Analysis shows a sensitivity of 0.78 and specificity of 0.87 with a cut-off of 3 points [45]. The PC-PTSD was delivered in Spanish to Latinx primary care patients, demonstrating cultural appropriateness [46]. The PC-PTSD showed strong psychometric properties in similar Latinx student populations. It was tested as a screening tool for middle-school aged students using school-based health centers, where 64% of the study sample were Latinx adolescents from schools in which, on average, 40% of students were ELL students [47]. In this study, authors found high sensitivity (100%) and specificity (83%) when using a score cut-off of 2 or more items, and a sensitivity of 67% and specificity of 97% when using a score cut-off of 3 or more items, with high negative predictive values (97% for a cut-off of 3 or more items) [47]. Among the present sample, Cronbach’s alpha is 0.71.

#### Internal resilience

Internal resilience was assessed with the Resilience Youth Development Module (RYDM) of the California Healthy Kids Survey (CHKS) [48], which is administered in select school districts as a tool to track student resilience across internal and external assets. We analyzed the 12-item internal assets scale, which is composed of four subscales: self-efficacy, empathy, problem solving, and self-awareness [28]. For self-efficacy, a sample item is “I can work out my problems.” For empathy, a sample item is “I try to understand what other people go through.” For problem-solving, a sample item is “I try to work out problems by talking or writing about them.” For self-awareness, a sample item is “I understand my moods and feelings.” Students provided responses ranging from “Not at all true,” to “Very much true.” The RYDM is scored by assigning each response option a value ranging from 1 to 4 and summing each item of the four subscales; higher scores indicate greater resilience. We measured a combined internal assets score (equal to the sum of all 12 items), in addition to a score for each subscale. Based on psychometric testing, the RYDM demonstrates construct validity and reliably assesses internal assets associated with positive youth development and student risk across racial and ethnic students, including Latinx students [48]. Psychometric testing of samples drawn from middle and high school students (grades 7, 9, and 11) across racial/ethnic groups identifying as Black, Chinese American, Mexican-American, and white demonstrated no significant differences in internal consistency among race/ethnicities [48]. Further, in a sample consisting of 37% youth identifying as Latinx, Furlong and colleagues found low variability in RYDM scores among ethnic groups (less than 1%) and some variability among genders (2.3%), however attributed most variation to individual student differences [28]. Based on student response distribution to the RYDM, Furlong and colleagues advocated for the use of the module with individual students by school psychologists or as a pretest-posttest evaluation [28]. Although Hanson and Kim, and Furlong did not report on the validity of measures for Latinx ELL students in their psychometric evaluations, the CHKS is available in Spanish and delivered to Latinx ELL students [28, 48, 49]. Within this sample, Cronbach’s alphas for the subscales associated with self-efficacy, empathy, problem solving, self-awareness, and the internal assets scores are 0.71, 0.81, 0.69, 0.75, and 0.85, respectively.

#### External resilience

External resilience typically refers to environmental supports (e.g. family, peer, and community supports), that support and meet the needs of youth, and help foster internal assets, leading to positive youth development and school outcomes [29]. The District assessed external resilience as the students’ perceived support at school, comprised of six items from the Caring Relationships and High Expectations scales of the RYDM that ask about support from a teacher or adult at the school [48]. This was consistent with Hanson and Kim’s (2007) recommendation that the six items of the RYDM Caring Relationships and High Expectations Scale be combined as one scale of “school support” based on factor analysis [48]. Each question starts with the prompt, “At my school, there is a teacher or some other adult” followed by a statement asking about support. An example of an item is, “At my school, there is a teacher or some other adult who tells me when I do a good job.” Each response, ranging from “Not at all true,” to “Very much true,” is assigned a value from 1 to 4 and the item scores are summed to create the scale; higher scores indicate greater perceived school support. This scale was validated for measuring external resilience among students based on psychometric testing by Hanson and Kim [48]. Additionally Furlong and colleagues found minimal variability among ethnic/racial groups of students [28]. Data available from WestEd from the 2019–2021 administration of the CHKS that included these six items demonstrates that 16.6–26.2% of the students sampled across the grades were categorized as “not proficient” in English, with Spanish the most common language spoken at home other than English among students surveyed [50]. Among the present sample, Cronbach’s alpha for the school support scale is 0.89.

### Statistical analysis

We first stratified by ELL-designation (ELL students vs. non-ELL students) and used means, standard deviations, frequencies, and percentages to describe student demographic and academic characteristics, risk for traumatic stress, and internal and external resilience. We tested for differences between students designated as ELL and non-ELL on these characteristics using chi-square tests and two-sample t-tests.

To examine the hypothesis that traumatic stress risk modifies the association between ELL-designation and resilience, we constructed a linear regression model regressing internal and external resilience on indicators for traumatic stress risk, ELL-designation and an interaction between these two factors. Models controlled for potential confounding factors that were identified by the significant chi-square tests and two-sample t-tests, and included: gender (male vs. female), grade (6–12), special education (Eligible vs. Not Eligible), attendance (Proficient/Advanced vs. Basic or Below), GPA (Passing vs. Not Passing), and birth country (United States vs. Other). Prior to fitting these models, we examined the distribution of each dependent variable within subsamples defined by the combination of these categorical variables and determined that the near normality and homogeneity of variance assumptions were likely reasonable (See supplemental Table 2). We then computed estimated means using a least squares means (LS means) approach and corresponding 95% confidence intervals. The LS means correspond to the estimated mean values using linear regression coefficients, controlling for gender, grade, special education eligibility, attendance, GPA, and birth country at values averaged over the levels of the controlling variables (i.e. for binary variables at 0.5, and for grade at 1/7 = 0.143). We identified interactions between traumatic stress risk and ELL-designation on internal and external resilience by testing for differences between LS means among students designated as ELL and non-ELL who were at high- and low-risk for traumatic stress, with an adjustment for making multiple comparisons across the four estimates implemented using the Tukey method. The inclusion of an interaction term for traumatic stress risk and ELL-designation allowed us to understand how differences in resilience between ELL and non-ELL students might differ in magnitude and direction for students at high- vs. low-risk for traumatic stress. Analyses and visualizations were conducted in SAS, version 9.4 and R, version 3.6.3.

An alternative analytical approach that was considered was the fitting of two separate regression models after stratifying based on traumatic stress risk. While this approach would have allowed for some added flexibility, the unified approach was ultimately selected for reasons of parsimony and the ability to test directly for differences between traumatic stress risk groups. Ultimately, the multiple linear regression modeling approach allowed us to control for numerous categorical and continuous variables and was thus preferred over a simpler two-way ANOVA formulation that was also considered.

## Results

We found significant differences between ELL and non-ELL students in demographic characteristics of gender, school-level (middle vs. high school), and birth country (Table 1). We also identified significant differences in academic characteristics, including special education eligibility, attendance, and GPA. Only 6% of non-ELL students were born outside of the United States compared to over one-quarter of ELL students (29%). Notably, almost one-third of ELL students received special education services (29%), compared to only 8% of non-ELL students, which as noted earlier, is similar to the district as a whole. A substantially higher proportion of non-ELL students had a passing GPA relative to ELL students, while a slightly elevated proportion of non-ELL students had Proficient/Advanced attendance compared to ELL students. ELL students reported significantly higher problem solving and perceived school support, while non-ELL students demonstrated significantly higher self-efficacy, empathy, and self-awareness.

**Fig. 1:**
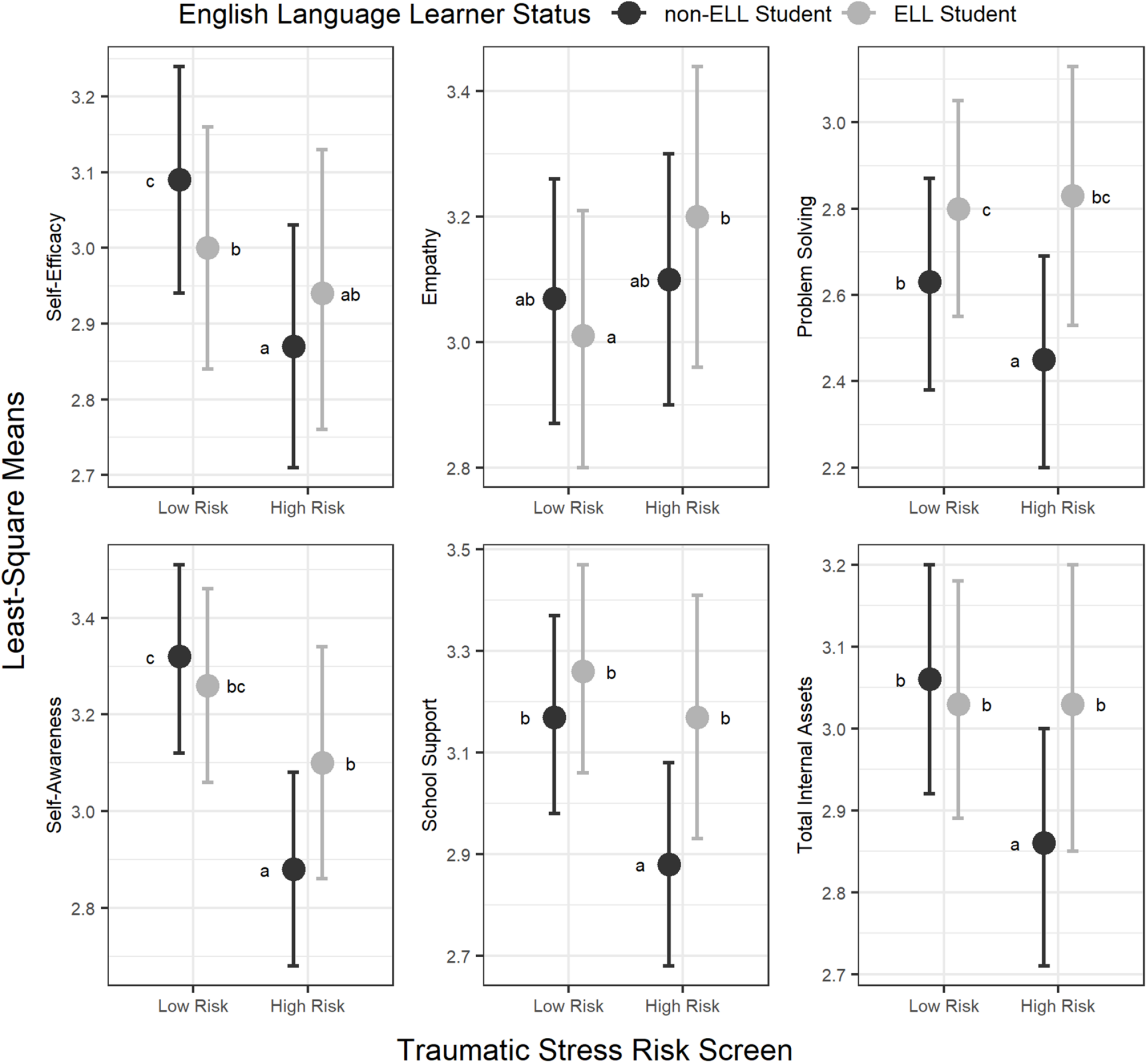
Resilience Among ELL and Non-ELL Latinx Students at High and Low Traumatic Stress Risk. *LS means that do not share the same letter differ significantly at the 0.05 level. Notes: Points indicate least square (LS) mean values, averaged over the levels of the controlling variables gender (male vs. female), grade (6–12), special education (eligible vs. not eligible), attendance (Proficient/Advanced vs. Basic or Below), GPA (passing vs. not passing), and birth country (US/other). Error bars represent the 95% confidence intervals of the respective LS mean. Means that do not share the same letter differ significantly at the 0.05 level. LS means that do not share the same letter differ significantly at the 0.05 level, with adjustment via the Tukey method for comparing four estimates

LS means for ELL and non-ELL students with high and low traumatic stress risk are presented in Table 2 and depicted graphically in Fig. 1. Among students with low traumatic stress risk, ELL students had significantly better problem solving, while non-ELL students had significantly better self-efficacy. Among students with high traumatic stress risk, ELL students had significantly better problem solving, self-awareness, perceived school support, and total internal assets.

**Table 2:** Resilience comparison among ELL and Non-ELL Latinx students at high and low traumatic stress risk

	ELL				Non-ELL			
	High Risk for Traumatic Stress		Low Risk for Traumatic Stress		High Risk for Traumatic Stress		Low Risk for Traumatic Stress	
	LS Mean	95% CL	LS Mean	95% CL	LS Mean	95% CL	LS Mean	95% CL
**Internal Resilience**								
Self-Efficacy	2.94	(2.76, 3.13)	3.00	(2.84, 3.16)	2.87	(2.71, 3.03)	3.09	(2.94, 3.24)
Empathy	3.20	(2.96, 3.44)	3.01	(2.80, 3.21)	3.10	(2.90, 3.30)	3.07	(2.87, 3.26)
Problem Solving	2.83	(2.53, 3.13)	2.80	(2.55, 3.05)	2.45	(2.20, 2.69)	2.63	(2.38, 2.87)
Self-Awareness	3.10	(2.86, 3.34)	3.26	(3.06, 3.46)	2.88	(2.68, 3.08)	3.32	(3.12, 3.51)
Total Internal Assets	3.03	(2.85, 3.20)	3.03	(2.89, 3.18)	2.86	(2.71, 3.00)	3.06	(2.92, 3.20)
**External Resilience**								
Perceived School Support	3.17	(2.93, 3.41)	3.26	(3.06, 3.47)	2.88	(2.68, 3.08)	3.17	(2.98, 3.37)

The results of all pairwise comparisons of LS means are displayed in Fig. 1. LS Means averaged over levels of controlling variables: grade (6–12), gender (male/female), special education status (yes/no), attendance (high/low), GPA (passing/non-passing), and birth country (US/other); all were treated as categorical variables

## Discussion

Latinx ELL students comprise an important and growing population of students in the U.S. As schools embrace ELL students and their families, it is critical to understand traumatic stress and resilience characteristics of Latinx ELL students to tailor services and promote academic and social-emotional outcomes. This study revealed significant general differences in internal and external resilience among Latinx ELL and non-ELL students that can inform school-based resilience and social-emotional interventions. First, we discuss the findings related to the central study questions regarding if resilience differs between ELL and non-ELL Latinx students, and if traumatic stress modifies the association between ELL designation and resilience, and then discuss implications of these findings for schools, school staff, and mental health clinicians working with Latinx ELL students.

Research Question 1: Does resilience differ between ELL vs. non-ELL Latinx students?

Our first research inquiry was to examine if resilience differed between ELL vs. non-ELL Latinx students. We found significantly higher problem solving and perceived school support among ELL students relative to non-ELL students. Non-ELL students reported significantly higher self-efficacy, empathy, and self-awareness. In the following section, we highlight the key findings of strengths of ELL students compared to non-ELL students.

### Problem-Solving among ELL students

Problem-solving is a form of executive control that is likely practiced daily among ELL students as they traverse new language, societal, system, and community norms, including navigating challenges of acculturation [14]. Research from ELL early childhood populations suggest that bilingual children exhibit higher levels of executive control and theory of mind understanding when immersed in environments where they must process in which language to communicate and how to interact with others [51].

Problem-solving is also an individual resilience protective factor, described by Benard and Slade as an internal asset that can be strengthened to help students cope with challenges, foster learning, and protect against risky behaviors, and is one of the most widely taught components in SEL and resilience interventions [29, 52, 53]. In their sociocultural model for Latinx youth, Blanco-Vega and colleagues describe how immigrant youth need coping skills to cope with the process of acculturation (including becoming aware of one’s ethnic identity, coping with parental expectation of adaptation, and being in situations that may bring conflict with cultural values) and argue for culturally adapted interventions that address these skills [14]. This finding of higher scores of problem-solving among Latinx ELL students in our study points toward both the utility of schools assessing for problem-solving skills among Latinx ELL students and providing interventions that continue to foster problem-solving among Latinx ELL students to promote resilience and support coping with challenges. One example of a culturally adapted program that addresses problem-solving is the *Jóvenes Fuertes (Strong Teens)* program, culturally adapted by Castro-Olivo and Merrell for Spanish-speaking recent immigrant students and found to improve social-emotional resiliency for middle and high school students [54, 55].

### Perceived school support for ELL students

We also found that ELL students in our study perceived a higher level of school support compared to non-ELL students. Perceived school support is an important protective factor associated with well-being, positive mental health outcomes, and improved academic outcomes [56, 57]. In Benard and Slades’ youth development framework, school support is fostered through caring relationships, participation opportunities, and conveying high expectations from school staff, which help cultivate student resilience [29]. However, past research has demonstrated lower rates of school support among minoritized youth, related to racism, implicit bias, and structural barriers affecting students’ ability to access supportive relationships [58, 59]. Our findings also differ from that of Motti-Stefanidi and colleagues, who in a longitudinal study of youth, found worse adaptation of Greek immigrant students compared to their non-immigrant peers, which they partially attributed to low educational support for students. These findings led authors to call for enhanced school supports and policies promoting positive public attitudes towards immigrants [60]. Indeed, ELL students in the U.S. may also have to cope with feelings of discrimination and acculturation stressors as they adapt to U.S. schools [14].

One possibility for the higher perceived school support among ELL students compared to non-ELL peers in our sample may be the increased support, time, and resources, including mental health resources, provided for ELL students, which is not provided to their non-ELL peers in this district. However prior studies have demonstrated mixed findings regarding time in ELL programs and resilience outcomes [36, 37]. Castro-Olivo and colleagues’ findings that longer time in ELL programs was related to lower social-emotional resiliency among secondary school Latinx ELL students heralded concerns that the social-emotional needs of these students were being undetected and unmet [37]. Han’s study of elementary school students—including Spanish speaking bilingual students—demonstrated the importance of school support in promoting social-emotional skills [36]. They found that school stability (lower teacher turnover and student and staff absenteeism) was associated with faster growth rates in teacher-rated learning approach, self-control, and interpersonal skills, and that a supportive teaching environment was associated with slower rates of problem behaviors among all students in the sample. Yet, Han also found that schools with more teachers and principals with English as a second language (ESL) experience rated student socioemotional growth as much slower compared to schools with principals and teachers with less ESL experience [36].

A sense of school belonging and school community involvement are protective factors for students; school belonging is associated with achievement motivation among both immigrant and non-immigrant Latinx youth [14, 61]. On a school-wide level, there are promising school models in the U.S. to increase supports for immigrant and ELL students through a whole-school approach. One such model is the “newcomer schools” model, which provides support to meet the social needs of student and families, and emotional wellbeing of students, including events that foster and celebrate immigrant identity [62]. These schools also provide staff professional development that build cultural understanding and promote cultural assets of students. Another model is the community schools model which focuses on providing preventative social services to support students and engage families through partnerships between the school and community partners, including services that support ELL students and their families [63]. More research is needed to determine if these types of school models enhance perceived school support for ELL students particularly.

Research Question 2: Does traumatic stress risk modify the association between ELL designation and resilience among Latinx students?

Our second research question inquired if traumatic stress risk modifies the association between ELL status and resilience among Latinx students. We found that problem-solving was higher among ELL students compared to their non-ELL peers, regardless of traumatic stress risk. Perceived school support was higher among ELL students relative to non-ELL students only in the high traumatic stress risk group. Similarly, ELL students also had significantly higher self-awareness and total internal assets relative to non-ELL peers only among the high traumatic stress group. In other words, when traumatic stress was high, ELL students displayed higher resilience across multiple areas of internal resilience (problem-solving, self-awareness, and total internal assets), and external resilience (school supports), when compared to non-ELL students. There were fewer significant differences between ELL and non-ELL when traumatic stress was low.

Overall, these study findings suggest that despite traumatic stressors experienced by Latinx ELL students, there are evident internal resilience strengths, and external resilience in the form of school support. This is consistent with decades of resilience research with youth experiencing traumatic experiences and structural vulnerabilities that demonstrate youths’ abilities for adaptations in the face of adversity [52], and earlier work demonstrating enhanced socio-emotional strengths among ELL students [36]. The outcome of higher perceived school support among Latinx ELL students with high traumatic stress, also validate the important contextual role of schools in addressing youth resilience as described by Suárez-Orozco and colleagues, Motti-Stefanidi and colleagues, and by the World Health Organization’s Health-Promoting Schools Framework [18, 40, 41]. Our findings align with Suárez-Orozco and colleagues’ integrative risk and resilience model for immigrant origin children and youth, in which schools are a microsystem that can promote positive outcomes among ELL and immigrant youth through culturally responsive teaching, supporting acculturative tasks, and promoting peer and teacher relationships [18]. Thus, our finding of higher perceived school support among ELL students with high traumatic stress risk—despite the stressors these students may experience—differs from prior studies and adds to the literature in this area. It is important to note that the district in which this study took place has a longstanding history of serving newcomer students and has school-based clinicians often trained in immigrant mental health, trauma, and culture. Although beyond the scope of this study, it is possible these findings point to the ELL programs at the schools providing social-emotional and trauma-informed supports, and potentially an awareness of the trauma needs of ELL students. Ultimately these findings demonstrate a need for further research in this area, including qualitative research with Latinx ELL students, to understand what contributes to higher perceived school support, particularly for ELL students with traumatic stressors.

Given concerns in the literature about the mental health needs of Latinx ELL students and the social emotional outcomes among students in ELL programs, our results that students with high-traumatic risk still demonstrated greater perceived school support in the form of a caring adult at school are promising [5, 19, 23, 37]. Our results also support a growing literature in the area of positive childhood experiences (PCEs), described as “safe, stable, nurturing relationships and environments” that can mitigate the impact of adverse childhood events (ACEs) [64]. PCEs occur across the domains of families, school, neighborhoods, and communities, with research indicating a buffering effect between increasing number of PCEs and youth mental health outcomes among adolescents exposed to childhood trauma. Although there is not yet a consensus on PCE measures, the existing frameworks include a component of protective and supportive adult relationships, including within schools [64]. Future research is needed in expanding the understanding of PCEs in ELL Latinx students who have experienced traumatic stress, including studies measuring PCEs in this population.

Finally, regarding the findings related to the resilience skills of self-efficacy, empathy, and self-awareness, these skills were found to be significantly higher among non-ELL Latinx students compared to ELL Latinx students. However, when examining the role of traumatic stress, there were differences among these associations. Namely, self-efficacy—the feeling that one can handle a situation [65]— was significantly higher among non-ELL students at low risk for traumatic stress, relative to the other three groups, suggesting a need to strengthen self-efficacy among students designated as ELL and/or with high traumatic stress. This is particularly important because self-efficacy is related to higher academic achievement outcomes among students [35]. Self-awareness was significantly higher among ELL students compared to non-ELL students with high traumatic stress risk and showed no differences among students with low traumatic stress risk. Although this is speculative, this finding could signify that ELL students with traumatic stress had greater understanding of their purpose and moods and feelings. Empathy showed no significant differences among ELL vs. non-ELL students when accounting for traumatic stress risk. All three of these skills are core components of universal resilience curriculum and social-emotional programs taught in schools [42]. Although findings differed based on traumatic stress risk, they signify it would be helpful to bolster these skills to ELL students, such as through universal resilience curricula, SEL programs tailored for ELL students such as Jóvenes Fuertes, and supports through ELL programs and classroom teachers [42, 54].

Overall Implications for Schools and Providers Working with Latinx ELL Students.

These findings add to the literature by demonstrating that Latinx ELL students exhibit considerable overall resilience, despite often experiencing high traumatic stress. These strengths despite adversity are important to convey to mental health providers, school clinicians, teachers, staff, and school administrators working with Latinx ELL students, and to inform resilience building interventions for all Latinx ELL students, particularly students exposed to traumatic events. Interventions for students with high traumatic stress should aim to reinforce resilience skills found to be higher for Latinx ELL students, relative to their peers, such as problem-solving and overall internal resilience, and to strengthen areas where Latinx ELL students did not differ significantly relative to their peers, such as self-efficacy and empathy. Although there are a range of school-based resilience programs found to be beneficial for youth most in need [53, 66], there are few that have been culturally adapted for Latinx ELL students and evaluated to determine the impact on both mental health and academic outcomes.

Finally, although only correlational, these differences in resilience and school supports could be related to factors that we did not study and warrant further research. There are protective factors we didn’t measure in our study that may contribute to increased perceived support and resilience among ELL students, such as support from religious or other community organizations and family factors specific to new immigrant families. As discussed below in limitations, studies, including qualitative research, could identify other protective factors, within the school setting, as well as in the family and broader community that promote resilience in this population.

## Limitations

This study has several limitations worth noting. Students included in the final analytical sample do not represent a random sample of all ELL and non-ELL students. As described previously, this study used a convenience sample, although available demographic characteristics are similar to that of the District. Further, students included in these analyses were clustered within schools and classrooms and this nested structure was not accounted for in the analyses. Methods for assessing ELL status and measuring academic characteristics relied on past-year academic data that may or may not reflect a student’s status at the time in which the survey was completed. In addition, ELL students were more likely to receive special education services. These types of services may contribute to greater bolstering of social supports and access to supportive staff. While all students in the analytical sample identified as Latinx, more detailed information about Hispanic origin (i.e. Puerto Rican, Guatemalan, etc.) was not incorporated into the analyses. Results may be largely driven by the findings among Latinx students of Mexican origin (the most well-represented subgroup across the district) and may not accurately reflect associations between factors like traumatic stress and resilience for other subgroups with different cultural identifications. Additionally, although the CHKS is administered in Spanish, to our knowledge the items have not been validated for Latinx ELL students, suggesting an area for future research.

To address these study limitations, it would be beneficial for future research studies to examine ELL status, academic outcomes, traumatic stress risk, and resilience factors across a district-wide and larger population of students so that the findings are more generalizable, rather than relying on a convenience sample of students from one district, along with analyses that account for classroom and school level clustering. As our findings were focused on Latinx ELL students of mostly Mexican background given this was the most prevalent background in our district, it would be beneficial to examine these characteristics among different Latinx subgroups. This would also be beneficial as public attitudes, policies, and school support for immigrant students—including undocumented and newcomer students—can vary across states [2].

Additionally, as mentioned earlier, we specifically examined external resilience at the level of supports provided by a teacher or adult at the school. However larger studies that also evaluate external resilience assets across the different ecological levels (e.g. family, neighborhood, religious and community agencies serving immigrant youth and families) in addition to school-level factors, would enhance understanding of the factors that promote resilience among Latinx ELL and non-ELL students and provide more information about the role of schools in relation to family and community level supports. Mixed methods studies utilizing qualitative interviews with students could also provide more contextual information about internal and external resilience factors among ELL students—as explained by students themselves—including a deeper understanding of the potential roles that schools, staff, and ELL services play in bolstering school support.

## Conclusion

This study addresses an important gap in the current literature, specifically, how Latinx ELL students differ in resilience characteristics from non-ELL students among students with high and low traumatic stress risk. In summary, we found that resilience differed between ELL vs. non-ELL Latinx students: ELL students reported significantly higher problem solving and perceived school support than their non-ELL peers. When examining the role of traumatic stress, ELL students with low traumatic stress had significantly better problem-solving compared to their non-ELL peers, while students with high traumatic stress demonstrated significantly better problem solving, self-awareness, total internal assets, and perceived school supports compared to their non-ELL peers. These findings indicate that Latinx ELL students may demonstrate resilience despite adversity. As schools continue to welcome Latinx ELL students, these findings also offer practical areas and implications across the domains of schools, policy, and future research.

### School-Based implications

As schools embrace trauma-informed and resilience-building approaches to address social and mental health inequities among students, these findings can inform services and school-based programs for Latinx ELL students to improve wellbeing, mental health equity, and academic outcomes [42, 43]. For educators and school staff working with Latinx ELL students, it is important to understand and highlight the strengths of this population despite adversity and continue efforts to strengthen school supports. These findings point to the need for culturally responsive and trauma-informed training for school staff working with Latinx ELL students. There are resources for educators and school staff to support ELL students who may have experienced trauma. For example, the U.S. Department of Education Newcomer Toolkit is a robust guide that includes information on the stressors ELL and newcomers may face, including trauma and acculturation stressors, and practical activities for supporting social-emotional learning and providing social emotional supports in the classroom and school-wide [67]. School educators and administrators can also draw on components of the “newcomer school models” to support ELL students and their families, such as partnering with community agencies, and hosting events that celebrate immigrant and cultural identity [62]. The internationals network is an organization that partners with school districts to provide supports to immigrant and newcomer students and has resources for schools and educators available on their website [68]. The findings also suggest a need for bolstering resilience characteristics in the classroom setting, including through classroom activities that enhance SEL skills such as self-efficacy specifically for Latinx ELL students [69].

For school mental health clinicians working with Latinx ELL students—particularly students exposed to traumatic experiences—being aware of these potential internal and external resilience assets—while building on them through a strengths-based approach—would be helpful when providing mental health services to students and their families. For school and district leadership, incorporating ways to measure resilience and risk among specialized student populations, including ELL students, can better inform understanding, programming of services, and policies, as well as incorporating school-wide policies that celebrate the strengths of Latinx ELL students.

### Policy

This study suggests that Latinx ELL students may benefit from ELL program support that includes social and emotional learning skills, especially for those who have experienced traumatic stress. Policymakers should support dissemination of ELL programs that integrate culturally informed and trauma-informed mental health resources, along with expanding funding for training schools and staff for how to best support ELL students, and community schools models focused on immigrant students.

### Research

Future studies can build upon these exploratory findings and reduce the risk of potential biases by using a random sampling approach, following students prospectively, and purposefully selecting additional validated measures to better elucidate the relationship between trauma and resilience among ELL students. Given the emerging research of PCEs, it would be helpful to measure PCEs to understand what factors may mitigate risk, including developing measures for the ELL population [70]. Finally, more studies are also needed to best determine how to tailor school efforts to bolster resilience skills among Latinx ELL populations within schools and the effect of these interventions on both mental health and academic outcomes, including the need to evaluate classroom level interventions adapted for Latinx ELL students.

## Electronic supplementary material

Below is the link to the electronic supplementary material.

Supplementary Material 1

## Data Availability

The data that support the findings of this study were accessed with a Data Use Agreement from the school district that restricts data sharing outside of this project. Thus, the data cannot be made publicly available. Inquiries can be directed to the research team at rijadimaghsoodi@mednet.ucla.edu, however the authors are not permitted to share the data. Any access to the data would be subject to district approval and a Data Use Agreement with the district.
