# Supplementary material for: Resilience and traumatic stress among Latinx english language learners: a cross-sectional study of students from an urban school district
Source: BMC Public Health. 2025 Jul 15;25:2458. doi: 10.1186/s12889-025-23105-4 (PMC12261741; doi:10.1186/s12889-025-23105-4)
Supplement: Supplementary file 1 — Supplementary Material 1 [file 12889_2025_23105_MOESM1_ESM.docx]

**Supplemental Table 1: Characteristics of ELL and Non-ELL Latinx Students at High and Low Risk for Traumatic Stress**

| **Student characteristics**  **(N = 4852)** | **ELL** | | **Non-ELL** | |
| --- | --- | --- | --- | --- |
|  | **High Risk for Traumatic Stress**  **(N = 217)** | **Low Risk for Traumatic Stress**  **(N = 585)** | **High Risk for Traumatic Stress**  **(N = 1069)** | **Low Risk for Traumatic Stress**  **(N = 2981)** |
|  | **N (%)** | **N (%)** | **N (%)** | **N (%)** |
| **Gender^***^** |  | | | |
| Female | 105 (48.39) | 250 (42.74) | 624 (58.37) | 1354 (45.42) |
| Male | 112 (51.61) | 335 (57.26) | 445 (41.63) | 1627 (54.58) |
| **Grade^***^** |  | | | |
| Middle School (6–8) | 121 (55.76) | 273 (46.67) | 363 (33.96) | 943 (31.63) |
| High School (9–12) | 96 (44.24) | 312 (53.33) | 706 (66.04) | 2038 (68.37) |
| **Special Education^***^** |  | | | |
| Not Eligible | 154 (70.97) | 415 (70.94) | 986 (92.24) | 2756 (92.45) |
| Eligible | 63 (29.03) | 170 (29.06) | 83 (7.76) | 225 (7.55) |
| **School Attendance^***^** |  | | | |
| Basic or Below | 79 (36.41) | 186 (31.79) | 348 (32.55) | 867 (29.08) |
| Proficient/Advanced | 132 (60.83) | 383 (65.47) | 715 (66.88) | 2088 (70.04) |
| Missing | 6 (2.76) | 16 (2.74) | 6 (0.56) | 26 (0.87) |
| **Grade point average (GPA)^***^** |  | | | |
| Passing | 60 (27.65) | 217 (37.09) | 553 (51.73) | 1650 (55.35) |
| Not Passing | 64 (29.49) | 161 (27.52) | 268 (25.07) | 697 (23.38) |
| Missing | 93 (42.86) | 207 (35.38) | 248 (23.20) | 634 (21.27) |
| **Birth Country**^1***^ |  | | | |
| United States | 156 (71.89) | 424 (72.48) | 1000 (93.55) | 2804 (94.06) |
| Mexico | 14 (6.45) | 36 (6.15) | 45 (4.21) | 114 (3.82) |
| El Salvador | 30 (13.82) | 77 (13.16) | 13 (1.22) | 33 (1.11) |
| Guatemala | 12 (5.53) | 29 (4.96) | 6 (0.56) | 18 (0.60) |
| Honduras | 2 (0.92) | 12 (2.05) | 1 (0.09) | 8 (0.27) |
| Other^2^ | 3 (1.38) | 7 (1.20) | 4 (0.37) | 4 (0.13) |
| ^*^p < 0.05, ^**^p < 0.01, ^***^p < 0.01 for Pearson chi-square test of independence across 4 categories defined by EL-status and traumatic stress risk  ^1^Chi-square test comparing United States vs. all other birth countries combined  ^2^Other includes: Brazil, Colombia, Costa Rica, Cuba, Nicaragua, Peru, Philippines, Puerto Rico, Venezuela | | | | |

**Supplemental Table 2: Descriptive Statistics for ELL and Non-ELL Students at High and Low Risk for Traumatic Stress**

|  | ***Variable*** | ***N*** | ***Mean*** | ***Std Dev*** | ***Lower Quartile*** | ***Median*** | ***Upper Quartile*** |
| --- | --- | --- | --- | --- | --- | --- | --- |
| **ELL High Risk for Traumatic Stress** | Self-Efficacy | 216 | 2.88 | 0.61 | 2.50 | 3.00 | 3.25 |
|  | Empathy | 216 | 3.14 | 0.69 | 2.67 | 3.33 | 3.67 |
|  | Problem Solving | 216 | 2.69 | 0.87 | 2.00 | 2.50 | 3.50 |
|  | Self-Awareness | 216 | 3.00 | 0.77 | 2.67 | 3.00 | 3.67 |
|  | Perceived School Support | 217 | 3.04 | 0.70 | 2.50 | 3.00 | 3.67 |
|  | Total Internal Assets | 216 | 2.94 | 0.55 | 2.58 | 3.00 | 3.33 |
| **ELL Low Risk for Traumatic Stress** | Self-Efficacy | 585 | 2.95 | 0.61 | 2.50 | 3.00 | 3.50 |
|  | Empathy | 585 | 2.99 | 0.74 | 2.33 | 3.00 | 3.67 |
|  | Problem Solving | 584 | 2.65 | 0.88 | 2.00 | 2.50 | 3.50 |
|  | Self-Awareness | 585 | 3.15 | 0.71 | 2.67 | 3.33 | 3.67 |
|  | Perceived School Support | 585 | 3.15 | 0.69 | 2.67 | 3.17 | 3.83 |
|  | Total Internal Assets | 584 | 2.96 | 0.57 | 2.50 | 3.00 | 3.38 |
| **Non-ELL High Risk for Traumatic Stress** | Self-Efficacy | 1068 | 2.83 | 0.57 | 2.50 | 2.75 | 3.25 |
|  | Empathy | 1068 | 3.18 | 0.72 | 2.67 | 3.33 | 3.67 |
|  | Problem Solving | 1068 | 2.43 | 0.92 | 1.50 | 2.50 | 3.00 |
|  | Self-Awareness | 1068 | 2.87 | 0.80 | 2.33 | 3.00 | 3.67 |
|  | Perceived School Support | 1069 | 2.87 | 0.76 | 2.33 | 3.00 | 3.50 |
|  | Total Internal Assets | 1068 | 2.86 | 0.53 | 2.50 | 2.88 | 3.25 |
| **Non-ELL Low Risk for Traumatic Stress** | Self-Efficacy | 2980 | 3.07 | 0.55 | 2.75 | 3.00 | 3.50 |
|  | Empathy | 2979 | 3.10 | 0.72 | 2.67 | 3.00 | 3.67 |
|  | Problem Solving | 2981 | 2.53 | 0.88 | 2.00 | 2.50 | 3.00 |
|  | Self-Awareness | 2981 | 3.27 | 0.66 | 3.00 | 3.33 | 4.00 |
|  | Perceived School Support | 2981 | 3.12 | 0.70 | 2.67 | 3.17 | 3.67 |
|  | Total Internal Assets | 2979 | 3.04 | 0.51 | 2.67 | 3.08 | 3.42 |
